# Quantitative CT-based structural alterations of segmental airways in cement dust-exposed subjects

**DOI:** 10.1186/s12931-020-01399-9

**Published:** 2020-05-29

**Authors:** Taewoo Kim, Hyun Bin Cho, Woo Jin Kim, Chang Hyun Lee, Kum Ju Chae, So-Hyun Choi, Kyeong Eun Lee, So Hyeon Bak, Sung Ok Kwon, Gong Yong Jin, Jiwoong Choi, Eun-Kee Park, Ching-Long Lin, Eric A. Hoffman, Sanghun Choi

**Affiliations:** 1 School of Mechanical Engineering, Kyungpook National University, 80 Daehak-ro, Buk-gu, Daegu, 41566 South Korea; 2 Department of Internal Medicine and Environmental Health Center, Kangwon National University Hospital, School of Medicine, Kangwon National University, Chuncheon, South Korea; 3 Department of Radiology, College of Medicine, Seoul National University, Seoul, South Korea; 4 Department of Radiology, College of Medicine, The University of Iowa, Iowa City, Iowa USA; 5 Department of Radiology, Research Institute of Clinical Medicine of Chonbuk National University–Biomedical Research Institute of Chonbuk National University Hospital, Jeonju, South Korea; 6 Department of Statistics, Kyungpook National University, Daegu, South Korea; 7 IIHR-Hydroscience and Engineering, The University of Iowa, Iowa City, Iowa USA; 8 Department of Medical Humanities and Social Medicine, College of Medicine, Kosin University, Busan, South Korea

**Keywords:** Airway narrowing, Wall thickening, Bifurcation angle, Stiffness of airway structure, Percent emphysema

## Abstract

**Background:**

Dust exposure has been reported as a risk factor of pulmonary disease, leading to alterations of segmental airways and parenchymal lungs. This study aims to investigate alterations of quantitative computed tomography (QCT)-based airway structural and functional metrics due to cement-dust exposure.

**Methods:**

To reduce confounding factors, subjects with normal spirometry without fibrosis, asthma and pneumonia histories were only selected, and a propensity score matching was applied to match age, sex, height, smoking status, and pack-years. Thus, from a larger data set (*N* = 609), only 41 cement dust-exposed subjects were compared with 164 non-cement dust-exposed subjects. QCT imaging metrics of airway hydraulic diameter (*D*_h_), wall thickness (WT), and bifurcation angle (*θ*) were extracted at total lung capacity (TLC) and functional residual capacity (FRC), along with their deformation ratios between TLC and FRC.

**Results:**

In TLC scan, dust-exposed subjects showed a decrease of *D*_h_ (airway narrowing) especially at lower-lobes (*p* < 0.05), an increase of WT (wall thickening) at all segmental airways (*p* < 0.05), and an alteration of *θ* at most of the central airways (*p* < 0.001) compared with non-dust-exposed subjects. Furthermore, dust-exposed subjects had smaller deformation ratios of WT at the segmental airways (*p* < 0.05) and *θ* at the right main bronchi and left main bronchi (*p* < 0.01), indicating airway stiffness.

**Conclusions:**

Dust-exposed subjects with normal spirometry demonstrated airway narrowing at lower-lobes, wall thickening at all segmental airways, a different bifurcation angle at central airways, and a loss of airway wall elasticity at lower-lobes. The airway structural alterations may indicate different airway pathophysiology due to cement dusts.

## Background

Dust exposure has been reported as a risk factor for pulmonary disease. For example, occupational dust exposure has been significantly associated with chronic obstructive pulmonary disease (COPD) [1]. Exposure to desert dust has been correlated with an increased risk of hospitalization for asthma [2]. An association between dust exposure and lung function has been reported via cytological and spirometry findings. In dusty areas near cement plants, the serum mercury level of blood samples was correlated with a decrease in the forced expiratory volume in 1 s (FEV_1_) and a risk of obstructive lung disease [3]. In addition, workers exposed to dust working in a cement factory were likely to have a decrease in peak expiratory flow [4]. However, the effects of environmental dust exposure on residents near cement plants have not been studied in detail. In this study, we hypothesize that environmental dust exposure by cements is associated with alterations of quantitative computed tomography (QCT)-based airway structural and functional metrics. Thus, QCT imaging-based variables are used to investigate structural and functional alterations due to dust exposure.

With respect to QCT imaging, few studies have investigated the effects of dust exposure on airway structure and lung function. For instance, coal and gold miners have been found to have a higher prevalence of emphysema compared with control groups [5]. The emphysema score measured by QCT has been associated with construction workers who are heavily exposed to asbestos [6] but not quartz and silica [7, 8]. Many previous studies have been limited regarding fully understanding the effects of dust exposure because they employed only one or a few imaging variables for a small number of subjects. More recently, Marchetti et al. [9] demonstrated that occupational dust-exposed subjects had a greater percentage of emphysema, percentage of air trapping, and wall area. The advanced post-processing of QCT imaging can reveal more airway structure features, such as airway luminal hydraulic diameter (*D*_h_), wall thickness (WT), and bifurcation angle (*θ*) in proximal airways, as well as parenchymal functional features, including air volume, tissue volume, the determinant of Jacobian (Jacobian), percent functional small airway disease (fSAD%), and percent emphysema (Emph%), through the image registration technique [10]. QCT metrics were able to classify clinically meaningful clusters of asthma [11].

With a comprehensive set of QCT imaging-based metrics, we aim to investigate unique features of airway structure and lung parenchymal function between subjects exposed to cement dust (dust-exposed: DE) and subjects with none or little exposure to cement dust (non-dust-exposed: NDE). The DE and NDE subjects were acquired at two different imaging sites, respectively. Both imaging sites collected two CT images for a subject at functional residual capacity (FRC) and total lung capacity (TLC). To minimize the intersite variability, we employed a fraction-threshold method [10, 12], when estimating parametric response map, i.e., fSAD% and Emph%. Next, to control the intersubject variability due to sex, age, height, smoking history, pack-years, and more, we employed a statistical method, i.e., propensity score matching method [13]. This allows for an objective comparison between two groups.

## Methods

### Study population

We employed 311 subjects with cement dust effects collected from Kangwon National University Hospital (KNUH) supported by a Korean research project called the Chronic Obstructive pulmonary disease in Dusty Areas near cement plants (CODA) cohort over approximately 10 years [3, 14, 15]. This project was designed to investigate the effect of cement-dust exposure on patients’ health near cement plants located in the Kangwon and Chungbuk provinces of South Korea considering the distance of cement plants and wind direction based on meteorological data from the National Institute of Environmental Research of the Ministry. The size of cement dust varies between 0.5 and 5 μm [16]. As control data, we employed 298 subjects with none or little exposure to cement dust collected from Chonbuk National University Hospital (CNUH) over 3 years [17]. The control subjects had normal findings on CT imaging, such as an absence of lung lesions or air-trapping, and no known history of lung disease or surgery. Both the KNUH and CNUH studies were approved by the Institutional Review Board at individual sites (KNUH 2019–06-007 and CUH 2016–03–020-005) and used a similar imaging protocol (Table 1).

**Table 1:** Scanners and scanning protocol used for non-dust-exposed subjects and dust-exposed subjects

	Non-dust-exposed subjects	Dust-exposed subjects
Institution	CNUH	KNUH
Scanner make	Siemens Definition Flash 128 slices	Siemens Definition AS 64 slices
Scan type	Spiral	Spiral
Rotation time(s)	0.5	0.5
Detector configuration	128 × 0.6 mm	64 × 0.6 mm
Pitch	1	1
Peak kilovoltage, kVp	120	140
mAs	110, Effective	100, Effective
Dose modulation	Care dose OFF	Care dose OFF
Reconstruction algorithm	B35f	B30f
Thickness (mm)	1	0.6
Iterative reconstruction	No selection	No selection

*CNUH* Chonbuk National University Hospital, *KNUH* Kangwon National University Hospital, *mAs* Milliamperage seconds

**Fig. 1:**
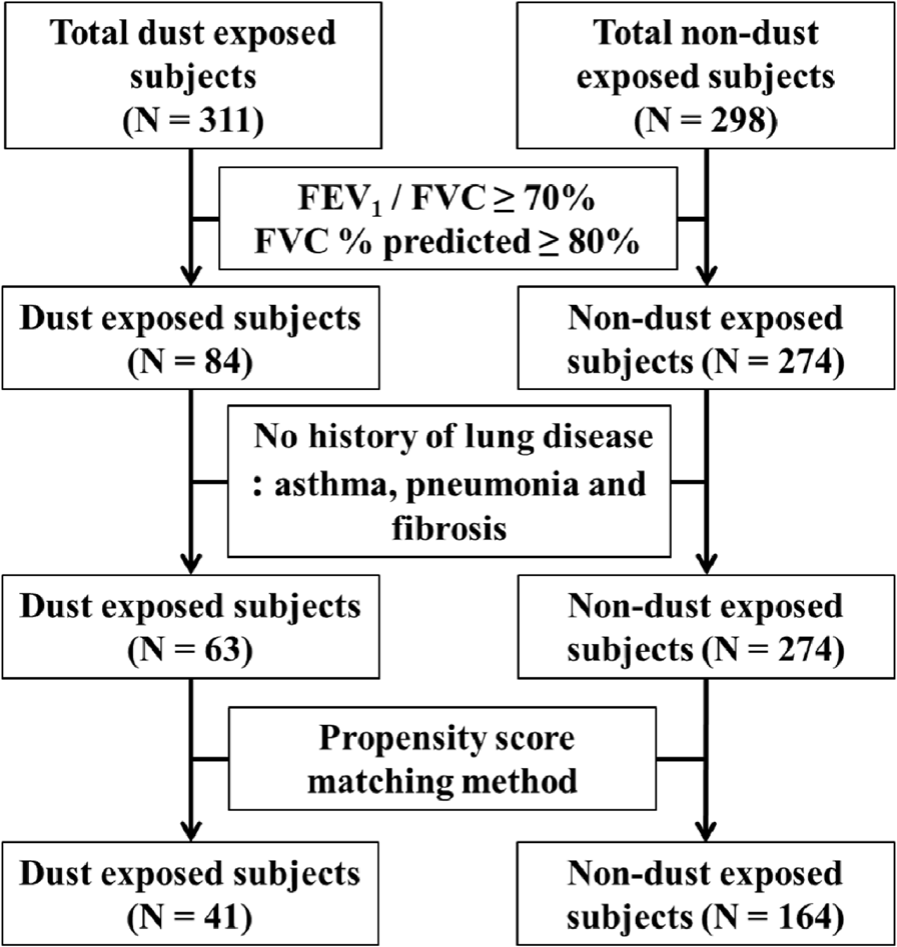
Flow chart of subject selection for dust-exposed subjects and non-dust-exposed subjects. FEV_1_, forced expiratory volume in 1 s; FVC, forced vital capacity

A flow chart for the subject selection procedure is provided in Fig. 1. To select subjects with normal lung function, we only included subjects with FEV_1_/forced vital capacity (FVC) ≥ 70% and FVC %predicted value≥80%. In addition, we excluded subjects with any prior diagnosis of fibrosis, asthma and/or pneumonia. This exclusion procedure allows for an objective comparison by eliminating confounding effects caused by pulmonary diseases such as fibrosis, asthma, pneumonia, and COPD. Then propensity score matching (PSM) method was applied for 63 DE and 274 NDE subjects, to reduce the confounding effects of age, sex, height, smoking history, and pack-years. See the subsection *Statistical analysis* for the PSM method. In this study, pulmonary function tests (PFTs) of DE and NDE subjects were performed according to the American Thoracic Society/European Respiratory Society guideline [18].

### QCT-based airway structure and lung function

In both TLC and FRC scans, we derived the luminal hydraulic diameter (*D*_h, TLC_ and *D*_h, FRC_), airway wall thickness (WT_TLC_ and WT_FRC_), and bifurcation angle (*θ*_TLC_ and *θ*_FRC_) using Apollo software 2.0 (VIDA Diagnostics, Coralville, Iowa, USA), along with an in-house post-process. The bifurcation angle was defined as an angle between two daughter branches of a proximal airway. *D*_h_, WT, and *θ* could be used to assess airway narrowing, wall thickening, and the alteration of branching structure, respectively.

**Fig. 2:**
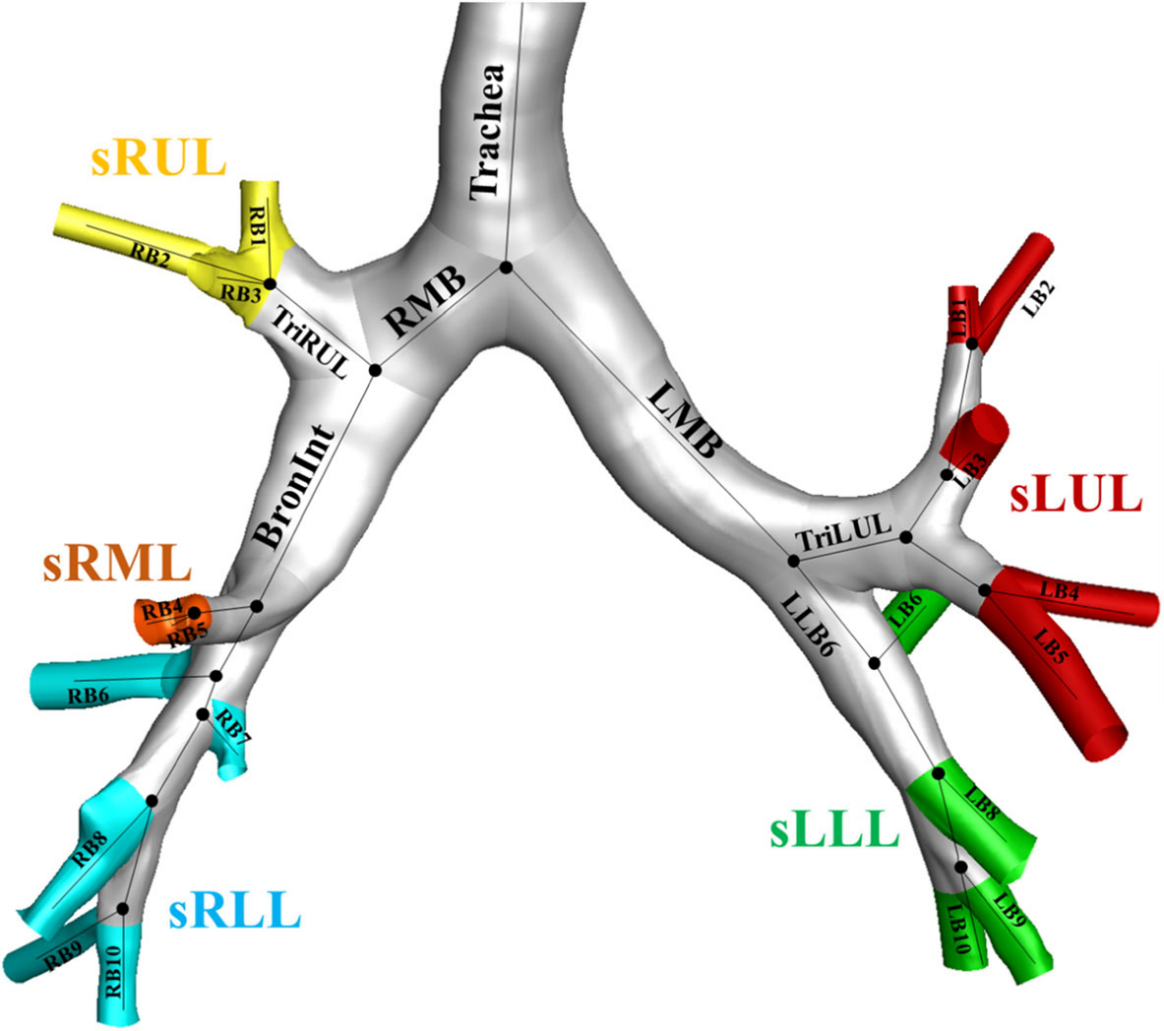
Labels of 26 segmental airways and 5 subgroups of lobes. BronInt, bronchus intermedius; LMB, left main bronchus; RMB, right main bronchus; sLLL, subgrouped left lower lobe including branches of LB6 and LB8 to LB10; sLUL, subgrouped left upper lobe including branches of LB1 to LB5; sRLL, subgrouped right lower lobe including branches of RB6 to RB10; sRML, subgrouped right middle lobe including branches of RB4 and RB5; sRUL, subgrouped right upper lobe including branches of RB1 to RB3; TriLUL, trifurcation of left upper lobe; TriRUL, trifurcation of right upper lobe

To assess the deformable features of segmental airways, the deformation ratio ε between TLC to FRC was computed as follows:

1 $$ {\varepsilon}^{\phi }\ \left(\%\right)=\frac{\phi^{TLC}-{\phi}^{FRC}}{\phi^{FRC}}\times 100, $$

where *ϕ* is any structural variable of *D*_h_, WT, and *θ*, so *ε*^*Dh*^, *ε*^WT^, and *ε*^*θ*^ were derived in this study. To measure the regional features of airways, structural variables were extracted from seven central airways and five subgroup regions. A detailed description of airway labeling is given in Fig. 2. The seven central airways included the trachea, right main bronchus (RMB), bronchus intermedius (BronInt), trifurcation of the right lower lobe (TriRUL), main bronchus (LMB), trifurcation of the left upper lobe (TriLUL), and LLB6. The five subgroup lobes included the right upper lobe (sRUL), right middle lobe (sRML), right lower lobe (sRLL), left upper lobe (sLUL), and left lower lobe (sLLL).

Functional variables included the air volumes at TLC and FRC, inspiratory capacity (IC), and Jacobian between TLC and FRC, respectively. In addition, Emph% and fSAD% were computed using an image registration technique [19]. To minimize the inter-center variability of Emph% and fSAD%, a fraction-threshold [10] method was used. Detailed formulations of these imaging variables are included in the references [10, 11, 20].

### Statistical analysis

We used the PSM method to reduce the bias between two groups of DE and NDE subjects. A propensity score is the predicted probability of belonging to the treatment group, and would be calculated for each subject in the study [13]. The propensity scores were estimated by multiple logistic regression analysis using the age and height variables stratified sex and smoking status. Matching was done using the Greedy 1–4 matching within a caliper, 0.2 times standard error of propensity scores. To validate PSM we used standardized difference, defined balance as an absolute value less than 10 [21]. PSM method was conducted using *SAS 9.4* software.

**Fig. 3:**
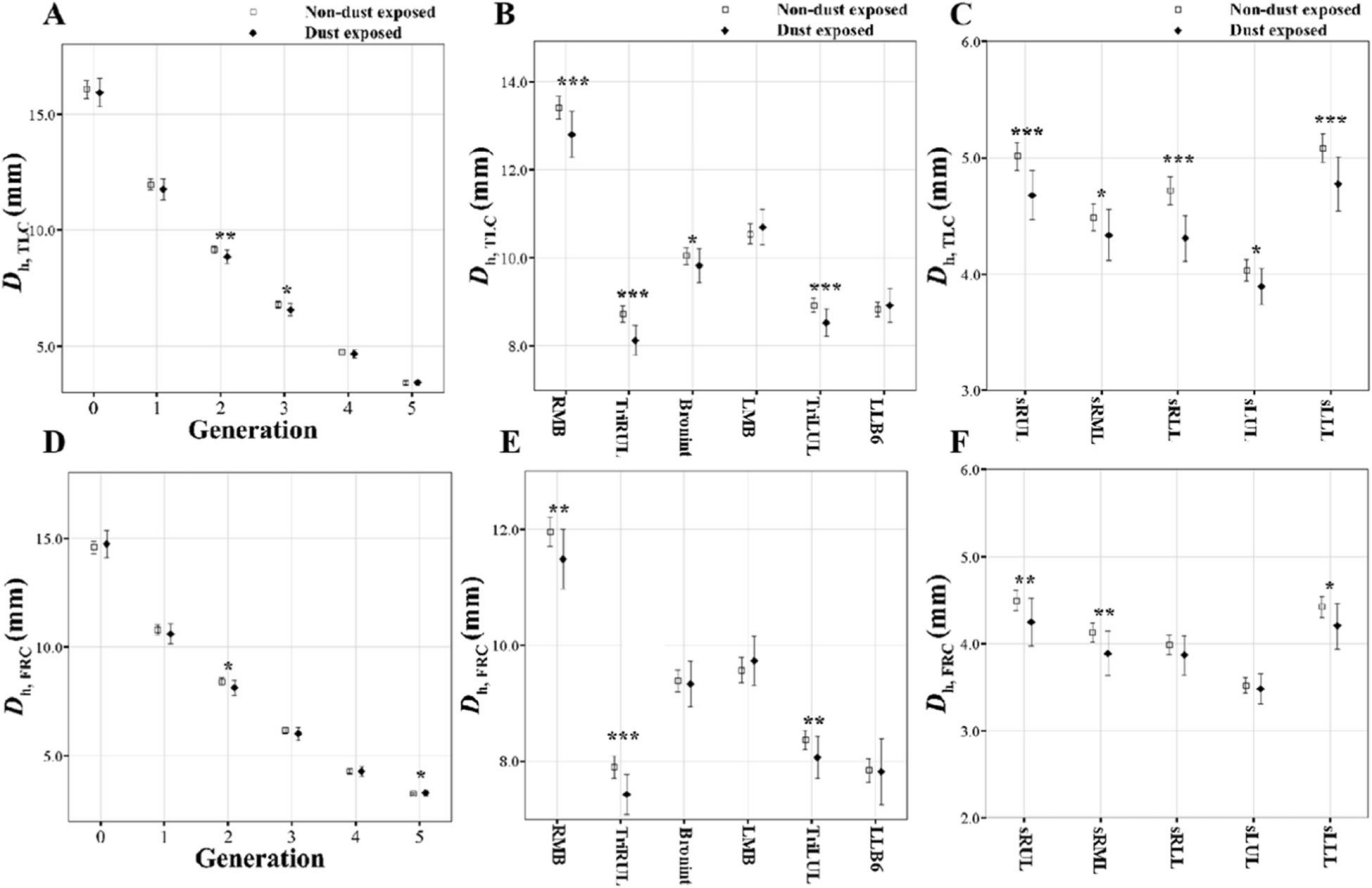
Comparison of luminal hydraulic diameter (*D*_h_) at TLC (**a**, **b**, and **c**) and FRC scans (**d**, **e**, and **f**) between non-dust-exposed subjects and dust-exposed subjects. Values are presented as mean (CI); *P < 0.05; **P < 0.01; ***P < 0.001. Generation zero is started from trachea. BronInt, bronchus intermedius; *D*_h_, hydraulic diameter; FRC, functional residual capacity; LMB, left main bronchus; RMB, right main bronchus; sLLL, subgrouped left lower lobe; sLUL, subgrouped left upper lobe; sRLL, subgrouped right lower lobe; sRML, subgrouped right middle lobe; sRUL, subgrouped right upper lobe; TLC, Total lung capacity; TriLUL, trifurcation of left upper lobe; TriRUL, trifurcation of right upper lobe

**Fig. 4:**
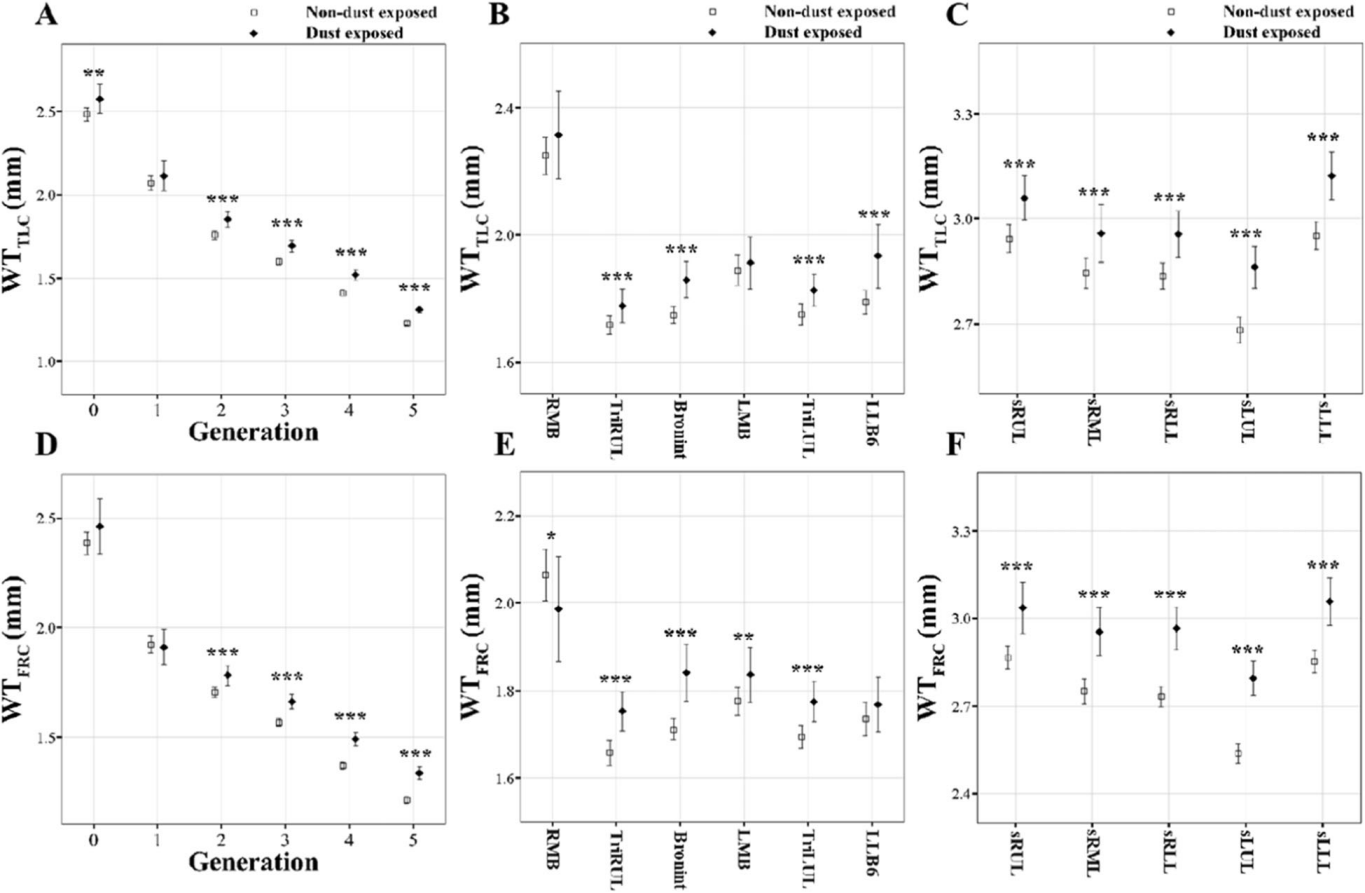
Comparison of wall thickness (WT) at TLC (**a**, **b**, and **c**) and FRC scans (**d**, **e**, and **f**) between non-dust-exposed subjects and dust-exposed subjects. Values are presented as mean (CI); *P < 0.05; **P < 0.01; ***P < 0.001. Generation zero is started from trachea. BronInt, bronchus intermedius; FRC, functional residual capacity; LMB, left main bronchus; RMB, right main bronchus; sLLL, subgrouped left lower lobe; sLUL, subgrouped left upper lobe; sRLL, subgrouped right lower lobe; sRML, subgrouped right middle lobe; sRUL, subgrouped right upper lobe; TLC, total lung capacity; TriLUL, trifurcation of left upper lobe; TriRUL, trifurcation of right upper lobe

**Fig. 5:**
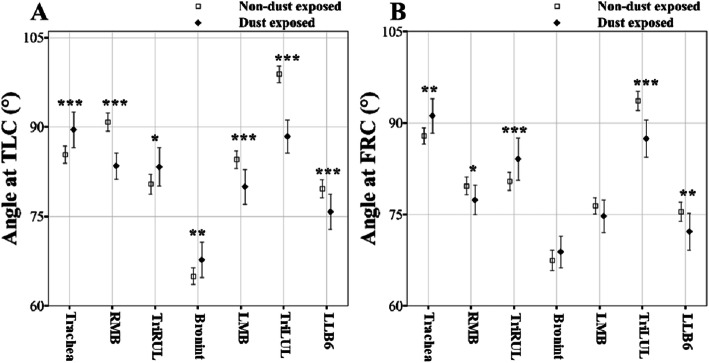
Comparison of airway bifurcation angle (*θ*) at TLC (**a**) and FRC (**b**) scans between non-dust-exposed subjects and dust-exposed subjects. Values are presented as mean (CI); *P < 0.05; **P < 0.01; ***P < 0.001. BronInt, bronchus intermedius; FRC, functional residual capacity; LMB, left main bronchus; RMB, right main bronchus; TLC, total lung capacity; TriLUL, trifurcation of left upper lobe; TriRUL, trifurcation of right upper lobe

**Fig. 6:**
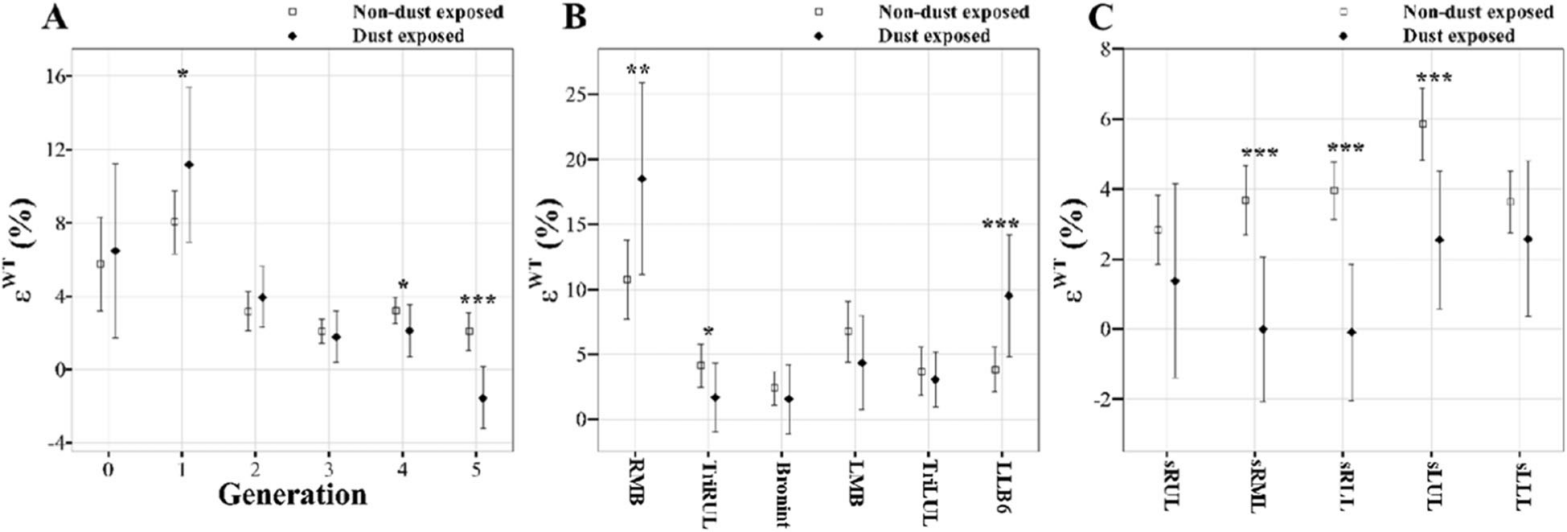
Comparison of deformation ratio of wall thickness (*ε*^WT^) between non-dust-exposed subjects and dust-exposed subjects. Values are presented as mean (CI); *P < 0.05; **P < 0.01; ***P < 0.001. Generation zero is started from trachea. BronInt, bronchus intermedius; FRC, functional residual capacity; LMB, left main bronchus; RMB, right main bronchus; sLLL, subgrouped left lower lobe; sLUL, subgrouped left upper lobe; sRLL, subgrouped right lower lobe; sRML, subgrouped right middle lobe; sRUL, subgrouped right upper lobe; TLC, total lung capacity; TriLUL, trifurcation of left upper lobe; TriRUL, trifurcation of right upper lobe

One sample t-tests were performed for the subjects matched by PSM method to compare the difference of QCT imaging-based metrics. The values are represented by means and the standard deviation (SD) in Tables 2, 3 and 4 and means and the confidence interval (CI) in Figs. 3, 4, 5 and 6. A significance level of *p* = 0.05 was chosen for a total of 135 comparison tests, resulting in a false discovery rate of 8.2%. Statistical analyses were conducted using *R* software.

**Table 2:** Propensity score matching before and after data using demographic (sex, age, height, smoking status, and pack-years) information for non-dust-exposed subjects and dust-exposed subjects

	Before propensity score matching					After propensity score matching (1:4)				
	NDE subjects		DE subjects		St.Diff.	NDE subjects		DE subjects		St.Diff.
	(*N* = 255)		(*N* = 63)			(*N* = 164)		(*N* = 41)		
	N	(%)	N	(%)		N	(%)	N	(%)	
Sex										
Male	99	(38.8)	45	(68.2)	61.6	80	(48.8)	20	(48.8)	0.0
Female	156	(61.2)	21	(31.8)		84	(51.2)	21	(51.2)	
Age										
mean ± std	51.0	±15.1	70.5	±7.5	±1.6	49.7	±15.4	70.0	±8.0	±1.7
Height										
mean ± std	161.4	±9.6	159.9	±10.1	±0.2	162.9	±9.7	157.9	±11.3	±0.5
Smoking										
Non/Former Smoking Participants	224	(87.8)	57	(86.4)	4.4	144	(87.8)	36	(87.8)	0.0
Current Smoking Participants	31	(12.2)	9	(13.6)		20	(12.2)	5	(12.2)	
Pack-years										
mean ± std	4.6	±13.2	14.7	±21.9	±0.6	4.4	±12.3	10.4	±22.7	±0.3
BMI										
mean ± std	–		–		–	24.4	±3.3	24.1	±2.8	0.372*

19 subjects were excluded by missing values of pack-years in *NDE* subjects. *NDE* Non-dust exposed, *DE* Dust exposed, *St. Diff.* Standardized differences; *, *P*-value obtained by one-sample t-test

**Table 3:** Comparison of deformation ratios of the bifurcation angle (*ε*^*θ*^) between non-dust-exposed subjects and dust-exposed subjects

Region	NDE subjects(*n* = 164)		DE subjects(*n* = 41)		*P* value
Trachea	−2.713	(7.232)	−1.713	(5.389)	0.191
RMB	14.51	(10.44)	8.172	(6.396)	< 0.001
TriRUL	1.290	(12.31)	4.194	(7.858)	< 0.05
BronInt	−2.171	(13.53)	−1.849	(11.07)	0.815
LMB	11.29	(10.40)	7.728	(12.39)	< 0.01
TriLUL	6.366	(9.857)	2.708	(9.361)	< 0.005
LLB6	6.505	(13.91)	5.084	(10.14)	0.266

Values are presented as mean (SD); *BronInt* Bronchus intermedius, *LMB* Left main bronchus, *RMB* Right main bronchus, *TriRUL* Trifurcation of right lower lobe, *TriLUL* Trifurcation of left upper lobe, *ε*^*θ*^ Deformation ratio of bifurcation angle

**Table 4:** QCT-based lung functions between non-dust-exposed subjects and dust-exposed subjects

QCT-based lung functions		NDE subjects (*n* = 164)		DE subjects (*n* = 41)		*P* value
TLC, liter		3.902	(1.030)	3.477	(0.970)	< 0.001
FRC, liter		2.163	(0.705)	1.977	(0.741)	< 0.05
IC, liter		1.794	(0.842)	1.543	(0.723)	< 0.005
Jacobian	Total	1.690	(0.364)	1.617	(0.348)	0.058
	LUL	1.601	(0.346)	1.558	(0.355)	0.258
	LLL	1.884	(0.452)	1.900	(0.469)	0.734
	RUL	1.581	(0.338)	1.440	(0.268)	< 0.001
	RML	1.465	(0.256)	1.391	(0.224)	< 0.005
	RLL	1.861	(0.436)	1.834	(0.414)	0.562
Emph%		0.017	(0.023)	0.010	(0.013)	< 0.005
fSAD%		0.087	(0.116)	0.080	(0.106)	0.554

Values are presented as mean (SD); *Emph%* Percent emphysema; *FRC* Functional residual volume, *fSAD%* Percent functional small airway disease, *IC* Inspiratory capacity, *TLC* Total lung capacity, *QCT* Quantitative computed tomography, *LLL* Left lower lobe, *LUL* Left upper lobe, *RLL* Right lower lobe, *RML* Right middle lobe, *RUL* Right upper lobe; The values are presented with absolute values in TLC, FRC, and IC, not predicted values

## Results

### Demographics information

Table 2 shows the demographic information for DE and NDE subjects in the before- and after- propensity score based matched data. The standardized differences of sex and smoking status were zero, indicating an exact matching. Standardized differences of continuous metrics, i.e., age, height, and pack-years were 1.4, 0.5, and 0.3, respectively. Note that the value of standardized difference smaller than 10 is considered as well-balanced. After matching the continuous metrics, body mass index (BMI) of two groups is also shown to be balanced.

### Segmental airways of dust-exposed subjects at TLC and FRC

Figure 3 shows the generational (left column) and regional (middle and right columns) differences in *D*_h_ between DE and NDE subjects from TLC scans (*D*_h, TLC_; top row) and FRC scans (*D*_h, FRC_; bottom row). Relative to NDE subjects, the *D*_h_, _TLC_ and *D*_h_, _FRC_ of DE subjects were smaller in the airways at RMB, TriLUL, and TriRUL (Fig. 3b and e). The significant difference of *D*_h_ was observed especially at all lobes in TLC (*P* < 0.05). Except for these airways, there were no or little statistical difference of the *D*_h_, _TLC_ and *D*_h_, _FRC_.

Figure 4 then shows the generational and regional differences of WT from TLC (top row) and FRC (bottom row) scans. DE subjects had increased WT in all regions except for RMB and LMB at the TLC scan and all regions except for LLB6 at the FRC scan. Consequently, WT was significantly increased from 2nd to 5th generation (*P* < 0.001), being different from *D*_h_. Compared with NDE subjects, the *θ*_TLC_, and *θ*_FRC_ of the trachea and TriRUL were increased, and the *θ*_TLC_ of RMB, LMB, TriLUL and LLB6 was decreased in DE subjects (Fig. 5a, b). Similarly, the *θ*_FRC_ of DE subjects were decreased in RMB, TriLUL, and LLB6 compared with that of NDE subjects (Fig. 5b).

**Fig. 7:**
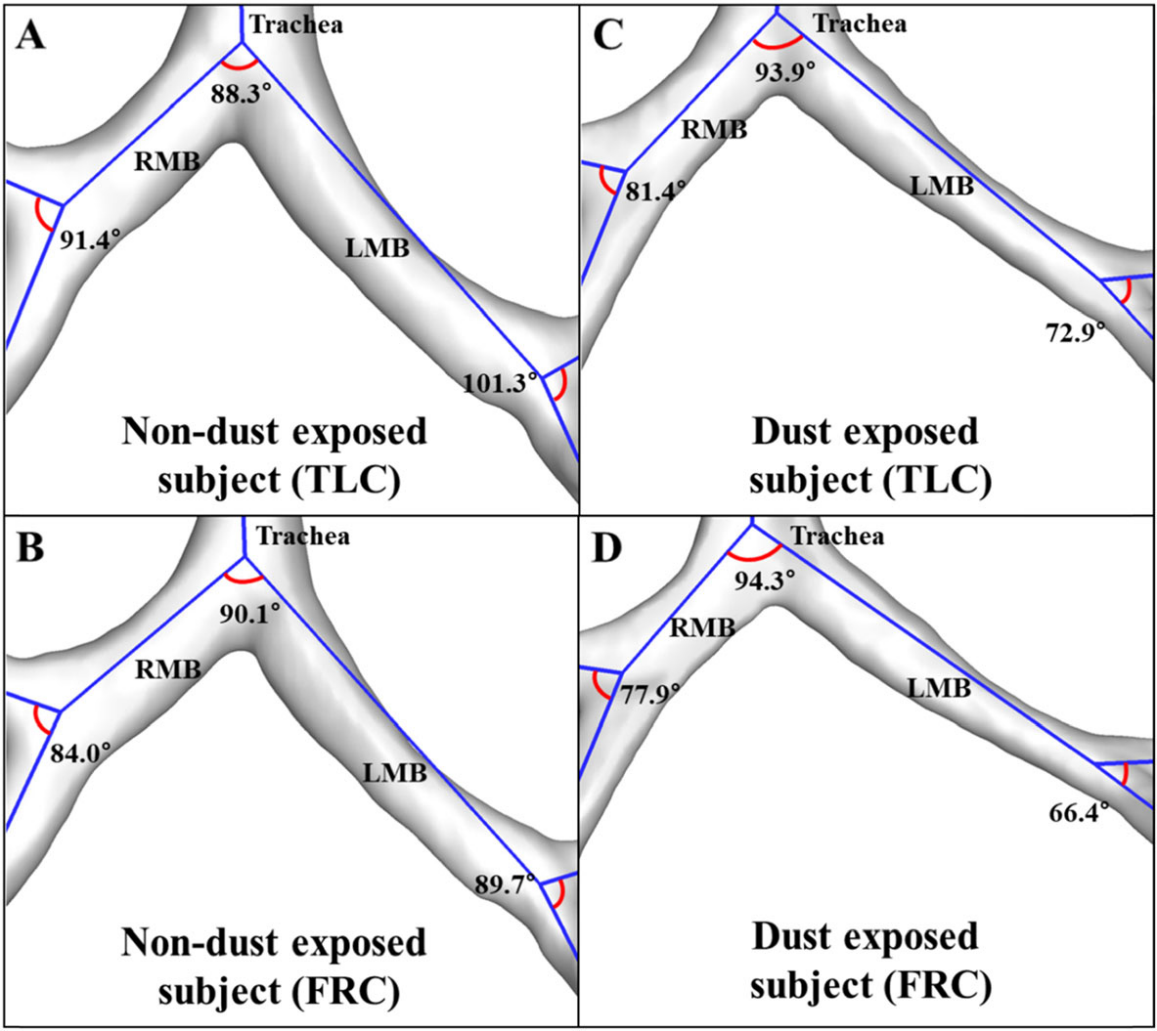
Bifurcation angle of airway in a non-dust-exposed subject at TLC (**a**) and FRC (**b**) scans and a dust-exposed subject at TLC (**c**) and FRC (**d**) scans. FRC, functional residual capacity; LMB, left main bronchus; RMB, right main bronchus; TLC, total lung capacity

Regarding the deformation ratio of *D*_h_ (ε^*Dh*^), there was no statistical difference between the two groups (not reported here). On the other hand, the *ε*^WT^ of the 4th–5th generations and all subgrouped lobes except sRUL and sLLL, namely segmental airways, were significantly smaller in DE subjects than NDE subjects (Fig. 6). Next, DE subjects had significantly smaller *ε*^*θ*^ in the RMB, LMB, and TriLUL (Table 3). Based upon WT and *θ*, DE subjects were found to have smaller deformations in bronchial structures when they breathe between TLC and FRC. The quantities possibly indicate an increase in airway stiffness of DE subjects. For instance, Fig. 7 supports this trend of bifurcation angle change at RMB between TLC and FRC in a DE subject (male; 74 years; BMI = 28) and an NDE subject (male; 67 years; BMI = 25).

### Functional features of dust-exposed subjects

**Fig. 8:**
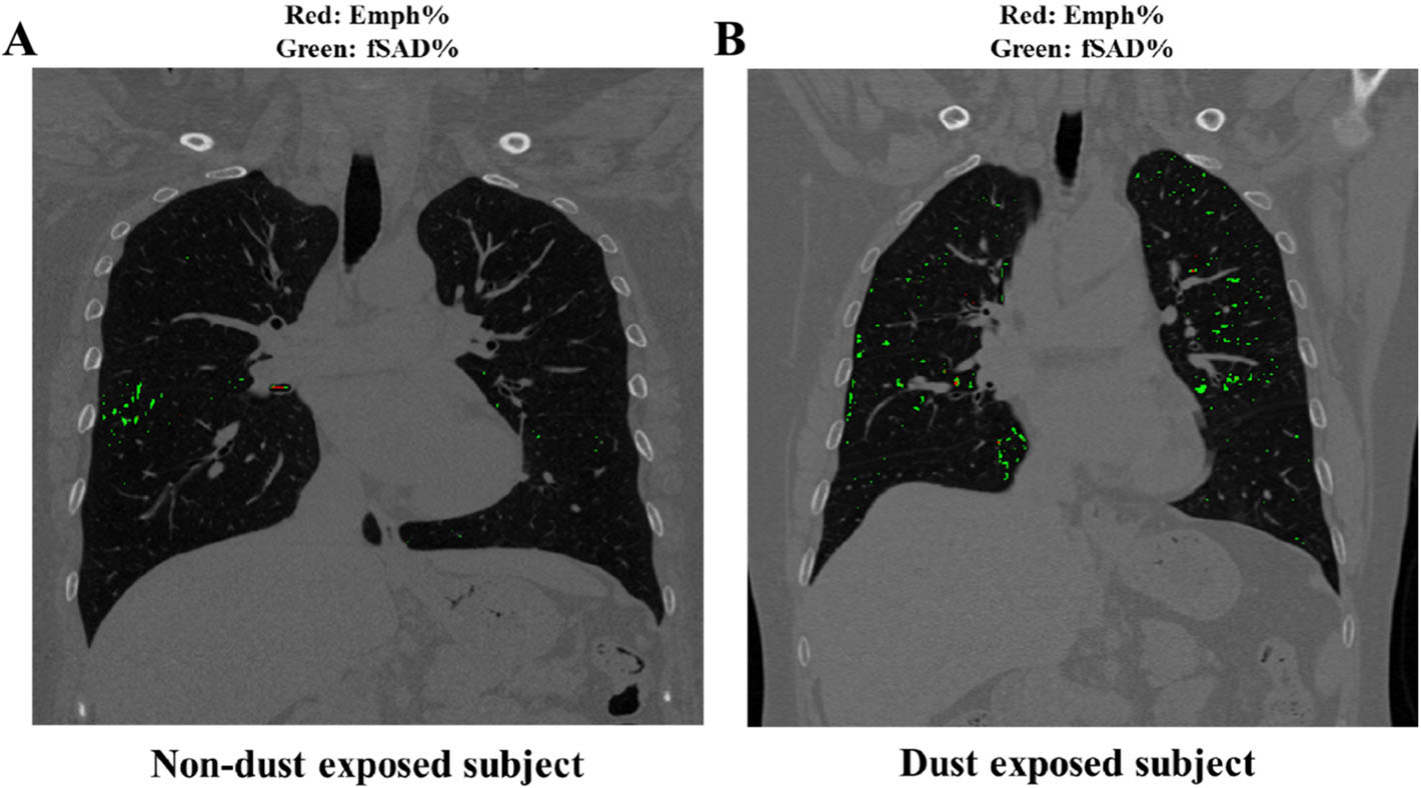
Parenchymal features of Emph% and fSAD% in a non-dust-exposed subject (**a**) and a dust-exposed subject (**b**)

Table 4 shows the differences of QCT-based functional metrics between NDE and DE subjects. The values of TLC, FRC, and IC were presented with absolute values, rather than % predicted values, because sex, age, and height of the data were already balanced by PSM method. Regarding air volumes, both TLC and FRC of DE subjects were smaller, and IC was also smaller in DE subjects than those of NDE subjects. The Jacobian indicating volume change ratio was decreased in DE subjects at right upper lobe and right middle lobe, consistent with IC. Emph% of DE subjects was lower than that of NDE subjects. However, fSAD% of DE subjects was not significantly different from that of NDE subjects. A figure was displayed to demonstrate parenchymal features of Emph% and fSAD% (Fig. 8).

## Discussion

In this study, with the aid of advanced QCT imaging analysis, we have investigated alterations of the airway structure and lung function at multiscale levels in subjects exposed to cement dust. Most similar studies [1–5, 8, 9] included patients with pulmonary diseases such as COPD and asthma, whereas for an objective comparison, this study excluded patients with pneumonia, asthma, and COPD to minimize confounding effects due to the pulmonary diseases. We also employed a robust statistical method of propensity score matching to control demographic confounders such as age, sex, height, smoking history, and pack-years. It has been known that imaging protocols between different centers are sensitive when estimating density-based imaging metrics such as Emph%, and fSAD% [22], whereas they are less sensitive on airway size parameters [20]. Therefore, we employed a fraction threshold method to compute the Emph% and fSAD%.

With sensitive QCT imaging metrics, we demonstrated that the airway structures of DE subjects had different features from those of NDE subjects. The DE subjects are characterized by phenotypes of airway narrowing (*D*_h_) at lower-lobes, wall thickening (WT) at all segmental airways, and alteration of branching structure (*θ*) at central airways. These findings were similarly observed in a previous study where individuals with occupational exposure had an increased airway wall thickness [9]. In the meantime, a multicenter study of former and current smokers using the multi-ethnic study of atherosclerosis (MESA) COPD data reveals that COPD subjects caused by mainly smoking have thinner airway walls [23]. The subjects in this study could be also progressed into COPD later, but these subjects exposed by cement dusts have thickened airway walls. A previous study has reported that exposure to cement dust leads to an increase in airway inflammation [24]. Thus, the distinguished phenotypes on airway walls are likely to indicate different airway pathophysiology.

A recent asthma study by Shim et al. [25] using severe asthma research program (SARP) data has demonstrated an association of airway lumen change between TLC and FRC with a corticosteroid treatment, but there were no investigations of wall thickness and branching angle changes between TLC and FRC. In this study, we computed strains for airway hydraulic diameter, wall thickness, and branching angle. To our best knowledge, this is the first effort of estimating strains at bronchial levels between TLC and FRC. As a result, the DE subjects were found to have the increased stiffness of wall thickness (ε^WT^) and bifurcation angle (ε^*θ*^) which could be affected by lung fibrosis and atelectasis, possibly due to the airway inflammation. In particular, the stiffened airways were likely to affect the prevention of airway deformation from FRC to TLC, sustaining the airway skeletal structure at FRC.

Regarding parenchymal functional variables (Table 4), lung volume at TLC, lung volume at FRC, IC, and Jacobian in DE subjects were smaller than NDE subjects. The decreased IC and Jacobian in DE subjects also could indicate a reduction of lung deformation. Especially, the reduction of Jacobian was found to be significantly correlated with ε^θ^ at RMB (Spearman test *R* = 0.416, *P* < 0.005). Based upon our analysis, we presume that the significantly reduced lung volume at TLC was caused by a reduced volume change (Jacobian). This is also possibly correlated with an increased stiffness of airways estimated by ε^WT^ and ε^θ^ (Table 3 and Fig. 6). In this study, fSAD% of DE subjects was similar with NDE subject, and Emph% of DE subjects was even lower than NDE subjects (Table 4). This is possibly due to the subgrouping by normal lung function, and also indicates that structural alterations of segmental airways begin earlier than parenchymal functional alterations.

Compared with lung functional metrics, airway structural variables provided very clear differences between the DE and NDE subjects. This implies that dust exposure due to cements was significantly associated with bronchial alterations in segmental scales rather than in parenchymal levels. As the size of cement dust ranges from 0.5 to 5 μm [16], cement particles may be deposited in segmental airways [24, 26, 27]. These features are different from the characteristics of cigarette smoke particles. Sahu et al. [28] demonstrated that the deposition rate of cigarette smoke particles was greater in parenchymal regions than in segmental regions due to the small size of the particles (ranging from 0.01 to 1 μm). A previous study found that smokers with normal spirometry were more susceptible to parenchymal alteration associated with the emphysema score [29, 30]. Whether the structural alterations observed here progress to parenchymal levels, leading to severe air-trapping and emphysema, has yet to be confirmed with a longitudinal study.

This study has several limitations. It was retrospectively designed by utilizing CT images collected at two respective sites. Thus, the findings obtained here were possibly influenced by intersite variability, such as scanner difference. However, as shown in Table 1, the two centers used the same scanner make (Siemens), same inspiratory maneuver (TLC), same expiratory maneuver (FRC), and similar reconstruction algorithms (B30f from KNUH and B35f from CNUH), so consistent regional attenuation, airway diameter, and wall thickness between the two groups are expected [31, 32]. In addition, the percent emphysema and percent fSAD were derived from the method using a fraction-threshold [10] that is a density variation-free method. Therefore, these results were not significantly influenced by scanner differences. In the previous study [20, 33], we already confirmed that different scanner had little confounding effect for QCT analysis with data derived from different sites. Furthermore, dust-exposed subjects could suffer from several pulmonary diseases such as interstitial lung disease and fibrosis which were not indicated by FEV_1_ and FVC. Therefore, it was better to include DLCO for the criterion when choosing subjects with normal lung function. Unfortunately, DLCO was not collected in this project, but we excluded any noticeable parenchymal diseases such as fibrosis, asthma, and pneumonia, so we believe that the current features in cement dust exposed subjects remained the same.

## Conclusions

In conclusion, with QCT imaging metrics, we demonstrated that DE subjects had unique features of airway structure, especially in segmental airways, compared with NDE subjects. In structural variables, DE subjects showed airway narrowing at lower-lobes, wall thickening at all segmental airways, a different bifurcation angle at central airways, and a loss of airway wall elasticity at lower-lobes compared with NDE subjects. Unlike segmental airways, parenchymal changes were relatively marginal at this stage for subjects with normal spirometry, which may be associated with the large size of cement dust. It has yet to be investigated if airway structural changes are associated with flow structure and particle distribution and deposition, so a future study with computational fluid dynamics is needed.

## Data Availability

Not applicable.

## Abbreviations

QCT: Quantitative computed tomography
*D*_h_: Hydraulic diameter
WT: Wall thickness
*θ*: Bifurcation angle
TLC: Total lung capacity
FRC: Functional residual capacity
COPD: Chronic obstructive pulmonary disease
FEV_1_: Forced expiratory volume in one second
Jacobian: Determinant of Jacobian
fSAD%: Percent functional small airway disease
Emph%: Percent emphysema
DE: Dust-exposed
NDE: Non-dust-exposed
KNUH: Kangwon national university hospital
CODA: Chronic obstructive pulmonary disease in dusty areas near cement plants
CNUH: Chonbuk national university hospital
FVC: Forced vital capacity
PSM: Propensity score matching
PFT: Pulmonary function test
BMI: Body mass index
*ε*^*Dh*^: Deformation ratio of *D*_h_
*ε*^WT^: Deformation ratio of WT
*ε*^*θ*^: Deformation ratio of *θ*
RMB: Right main bronchus
LMB: Left main bronchus
RUL: Right upper lobe
RML: Right middle lobe
RLL: Right lower lobe
LUL: Left upper lobe
LLL: Left lower lobe
BronInt: Right intermediate bronchus
TriLLB: Trifurcation of left lower lobe
sRUL: Sub-grouped right upper lobe with branches of RB1 to RB3
sRML: Sub-grouped right middle lobe with branches of RB4 to RB5
sRLL: Sub-grouped right lower lobe with branches of RB6 to RB10
sLUL: Sub-grouped left upper lobe with branches of LB1 to LB5
sLLL: Sub-grouped left lower lobe with branches of LB6, and LB8 to LB10
IC: Inspiratory capacity
SD: Standard deviation
CI: Confidence interval
MESA: Multi-ethnic study of atherosclerosis
SARP: Severe asthma research program
